# Correction: Microglia are essential for tissue contraction in wound closure after brain injury in zebrafish larvae

**DOI:** 10.26508/lsa.202403129

**Published:** 2024-11-25

**Authors:** Francois El-Daher, Stephen J Enos, Louisa K Drake, Daniel Wehner, Markus Westphal, Nicola J Porter, Catherina G Becker, Thomas Becker

**Affiliations:** 1 Centre for Discovery Brain Sciences, University of Edinburgh Medical School: Biomedical Sciences, Edinburgh, UK; 2 Center for Regenerative Therapies Dresden at the TU Dresden, Dresden, Germany; 3 Max Planck Institute for the Science of Light, Erlangen, Germany; 4 Max-Planck-Zentrum für Physik und Medizin, Erlangen, Germany; 5 Cluster of Excellence Physics of Life, TU Dresden, Dresden, Germany

## Abstract

Although in humans, the brain fails to heal after an injury, young zebrafish are able to restore tissue structural integrity in less than 24 h, thanks to the mechanical action of microglia.

Article: El-Daher F, Enos SJ, Drake LK, Wehner D, Westphal M, Porter NJ, Becker CG, Becker T (2024 Oct 17) Microglia are essential for tissue contraction in wound closure after brain injury in zebrafish larvae. Life Sci Alliance 8(1): e202403052. doi: https://doi.org/10.26508/lsa.202403052. PMID: 39419547.

Note from the authors:

In this correction, we have updated the citations and reference list in the Materials and Methods section to rectify inaccuracies in the original publication. Specifically, we corrected the author names and publication years for several references to ensure proper attribution and accuracy. These changes do not affect the results or conclusions of the study but are made to maintain the integrity and reliability of the scientific record.

## Materials and Methods

### Fish husbandry

All zebrafish lines were kept and raised under standard conditions (Westerfield, 2000) and all experiments were approved by the UK Home Office (project license no.: PP8160052) or according to German animal welfare regulations with the permission of the Free State of Saxony (project license no.: TVV36/2021). Following the guidelines of the 3Rs, we only used larvae aged up to 5 dpf. For experimental analyses, we used larvae of either sex of the following available zebrafish lines: Tg(Xla.Tubb:DsRed)^zf148^ (Peri & Nüsslein-Volhard, 2008); Tg(betaactin:utrophin-mCherry)^e119^ (Compagnon et al, 2014); Tg(h2a.F/Z:GFP)^kca6^ (Pauls et al, 2001) (referred to as Tg(h2a:GFP)); Tg(mpeg1.1:GFP)^gl22^ (Ellett et al, 2011); Tg(mpeg1.1:mCherry)^gl23^ (Ellett et al, 2011); Tg(irf8)^st95^ (Shiau et al, 2015); Tg(elavl3:MA-mKate2)^mps1^ (Tsata et al, 2021). The Tg(her4.3:GFP-F)^mps9^ transgenic zebrafish line has been previously described by Kolb et al (2023) and was established using the DNA constructs and methodology described below. If necessary, larvae were treated with 100 M Nphenylthiourea (PTU) to inhibit melanogenesis. All chemicals were supplied by Sigma-Aldrich unless otherwise stated.

### Generation of Tg(her4.3:GFP-F) transgenic fish

To create the donor plasmid for generation of her4.3:GFP-F transgenic zebrafish, the sequence coding for the membrane-localised GFP (EGFP fused to farnesylation signal from c-HA-Ras) was amplified from the pEGFP-F vector (Clonetech) using oligos 5′-TTATTTATCGATCCACCATGGTGAGCAAGGGC-3′ and 5′-TTTATTATCGATTCAGGAGAGCACACACTTGCAGCT-3′ and cloned downstream of the her4.3 (previously known as her4.1) zebrafish promoter (Yeo et al, 2007). Transgenic fish were established by injection of 40 pg of the donor plasmid together with mRNA of the Tol2 transposase into one-cell embryos (Suster et al, 2009).

### gRNA injections

The gRNAs were injected into the yolk at the one-cell stage of development. The injection mix was prepared on the morning of injections. The mix consisted of 1 liter Cas9 protein (M0369M; BioLabs), 1 liter Fast Green FCF dye (235345-9; Sigma-Aldrich), 1 liter 250 ng/liter SygRNA-tracr (TRACRRNA05N; Sigma-Aldrich), 1 liter gRNA, and 1 liter nuclease-free water. When two gRNAs were co-injected, the nuclease-free water was substituted with the second gRNA. After mixing gRNAs and tracr (and water if using), the mixture was heated to 95 degrees for 5 min and then kept on ice for 20 min. After this, the Cas9 and dye are added, and the mixture is again heated to 27 degrees for 10 min. For every experiment, two injection mixtures were made, one with the gRNA of interest and one with a control gRNA (5′-TTACCTCAGTTACAATTTAT-3′). lcp1 was targeted with a gRNA (5′-GAACCCGGUACCCCGGCAGA-3′) as previously published (Keatinge et al, 2021).
